# Evaluating the Efficacy and Safety of 48-Week Low-Dose Dienogest Administration in Patients With Dysmenorrhea Caused by Endometriosis: Protocol for a Randomized, Open-Label, Parallel-Group Trial

**DOI:** 10.2196/66246

**Published:** 2025-05-13

**Authors:** Kyoko Kikuno, Ryuta Asada, Takuma Ishihara, Yoshimasa Bomoto, Saki Murase, Yoko Ueda, Tomomi Shiga, Yoh Hayasaki, Tatsuro Furui, Satoko Matsuzaki, Masahiko Takemura, Kazutoshi Matsunami, Makoto Kubo, Naoki Ito, Masanori Isobe, Ken-ichirou Morishige

**Affiliations:** 1 Department of Obstetrics and Gynecology Gifu University Medical School Hospital Gifu Japan; 2 Innovative and Clinical Research Promotion Center Gifu University Medical School Hospital Gifu Japan; 3 Department of Obstetrics and Gynecology Osaka General Medical Center Osaka Japan; 4 Department of Obstetrics and Gynecology Matsunami General Hospital Gifu Japan; 5 Department of Obstetrics and Gynecology Hashima City Hospital Gifu Japan; 6 Department of Obstetrics and Gynecology Chuno Kosei Hospital Gifu Japan

**Keywords:** endometriosis, dysmenorrhea, dienogest, clinical trial, open-label

## Abstract

**Background:**

The treatment of endometriosis includes analgesics, hormone therapy, and surgery. Even after surgical removal of endometriotic lesions, the risk of recurrence remains high once the normal menstrual cycle resumes. Therefore, long-term hormone therapy is essential to prevent recurrence. Among hormonal treatments, low-dose estrogen progestin preparations are not recommended for patients older than 40 years due to the increased risk of thrombotic side effects. In contrast, dienogest does not carry a thrombotic risk, making it a suitable option for older patients. Although dienogest requires long-term administration until menopause in patients with endometriosis, data on its long-term efficacy and potential adverse effects remain limited. In particular, comparative studies assessing the safety and effectiveness of long-term use of dienogest at different doses (1 mg/day vs 2 mg/day) have not been conducted, highlighting the need for further investigation.

**Objective:**

The purpose of this study is to investigate the efficacy and the incidence of adverse events of dienogest 1 mg/day after 48 weeks in patients with dysmenorrhea due to endometriosis, compared with dienogest 2 mg/day.

**Methods:**

This randomized, open-label, parallel-group, dose-comparison, multicenter trial follows the SPIRIT (Standard Protocol Items: Recommendations for Interventional Trials) 2013 guidelines and is conducted at 6 centers in Japan. Participants are randomly assigned in a 1:1 ratio to receive either dienogest 1 mg/day or 2 mg/day. The drug is administered for 48 weeks, and its therapeutic effects and side effects are evaluated. Hospital visits include the use of questionnaires, vital sign measurements, imaging studies (magnetic resonance imaging and ultrasound), blood tests, and bone density assessments. The primary endpoint is the change in the pain visual analog scale (VAS) score from baseline to 48 weeks. The VAS is a 10 cm horizontal scale where 0 cm represents no pain and 10 cm represents the maximum imaginable pain; participants indicate their pain level on the scale, and the change is analyzed over time. The target sample size is 88, determined with a noninferiority margin based on existing literature. The protocol was approved by the Nagoya City University Hospital Clinical Research Review Board. Findings will be presented at academic conferences and published in peer-reviewed journals.

**Results:**

Currently, data collection is ongoing. The first participant was enrolled in August 2021. As of March 22, 2025, a total of 88 participants had been enrolled in this clinical trial.

**Conclusions:**

This is the first trial to compare efficacy and safety between 1 mg/day and 2 mg/day of long-term dienogest use in patients with dysmenorrhea caused by endometriosis. Combining diagnostic imaging with patient questionnaires and blood tests allows the determination of efficacy against endometriosis itself.

**Trial Registration:**

Japan Registry of Clinical Trials jRCTs041210016; https://jrct.mhlw.go.jp/en-latest-detail/jRCTs041210016

**International Registered Report Identifier (IRRID):**

DERR1-10.2196/66246

## Introduction

### Background

#### Endometriosis and Dysmenorrhea

Dysmenorrhea is characterized by menstrual pain, nausea, headaches, and related symptoms. One of its primary causes, endometriosis, is an estrogen-dependent disease affecting sexually mature women. This condition significantly reduces quality of life and imposes a substantial socioeconomic burden [1,2].

The incidence of endometriosis has increased due to delayed childbirth and declining birth rates. Hormonal and anatomical changes associated with menstruation and pregnancy significantly influence the risk of endometriosis. Research indicates that nulliparous women with an earlier age at menarche and shorter menstrual cycles are at the greatest risk due to increased lifetime menstrual exposure. Conversely, among parous women, both higher parity and longer lifetime duration of lactation are associated with a decreased risk of endometriosis. Given the current societal trends of delayed marriage and declining birth rates, it is anticipated that the prevalence of endometriosis will continue to rise in the coming years [3,4].

Although the exact etiology and pathogenesis of endometriosis remain unclear, no effective preventive measures exist. Endometriosis can begin at menarche and persist until menopause [5-7], often requiring long-term treatment. Surgical intervention may be necessary, but estrogen exposure in premenopausal patients increases recurrence risk, necessitating prolonged postoperative hormone therapy.

#### Hormonal Treatments for Endometriosis

Hormonal treatments include gonadotropin-releasing hormone agonists, low-dose estrogen progestin therapy, dienogest, and the levonorgestrel-releasing intrauterine system.

Gonadotropin-releasing hormone agonists suppress ovulation, inducing a hypoestrogenic state and alleviating symptoms. However, their use is limited to 6 months due to secondary hypoestrogenic side effects [8]. A low estrogen state, also referred to as hypoestrogenic symptoms, is characterized by significantly reduced circulating estrogen levels. This hormonal reduction can affect various bodily functions, including menstrual regulation, bone metabolism, and cardiovascular health. Common hypoestrogenic symptoms include hot flashes, night sweats, vaginal dryness, mood changes, and decreased bone density.

Levonorgestrel-releasing intrauterine system provides sustained annual efficacy without discontinuation risks but may be more difficult to use in patients with uterine fibroids, adenomyosis, or uterine deformities due to expulsion risks [9].

Low-dose estrogen progestin therapy is associated with an increased risk of thromboembolism, making it less favorable for patients aged 40 years or older [10].

#### Role of Dienogest in Endometriosis Management

Dienogest is a progestin with a lower risk of thromboembolism, making it suitable for patients older than 40 years [11]. It was first approved in Japan in 2007 for the treatment of endometriosis at a dose of 2 mg/day (administered as 1-mg tablets taken twice daily). In 2020, a lower dose of 1 mg/day (administered as 0.5-mg tablets taken twice daily) received approval for the treatment of dysmenorrhea.

However, dienogest 0.5-mg tablets are available only in Japan, and comprehensive knowledge regarding their pharmacological characteristics and potential side effects remains insufficient. In addition, data on the long-term efficacy of dienogest and the side effects associated with low estrogen symptoms are limited [12-14].

#### Consideration of Pharmacological Mechanisms

The therapeutic effects of dienogest on endometriosis are believed to result from multiple mechanisms. Harada et al [15] reported that dienogest reduces estrogen levels, thereby alleviating symptoms of estrogen-dependent endometriosis. In addition to estrogen suppression, dienogest demonstrates high selectivity for progesterone receptors [16] and exhibits antiproliferative activity in isolated human endometrial cells [17]. Furthermore, it has been shown to reduce the proliferative activity of the endometrium in a dose-dependent manner at doses of 0.5 mg or higher [18]. These findings suggest that dienogest’s efficacy may stem not only from lowering serum estradiol levels but also from directly inhibiting endometrial cell proliferation [15].

It has been reported that the use of 0.5 mg tablets results in serum estrogen levels comparable to those of a placebo [19]. However, their efficacy in treating dysmenorrhea has already been demonstrated. If this study shows that 0.5-mg tablets are as effective as 1-mg tablets in treating endometriosis, it may provide new insights suggesting that mechanisms beyond estrogen suppression contribute to the improvement of endometriosis.

The objective of this study is to evaluate the long-term efficacy and safety of low-dose dienogest (0.5 mg/day) compared to the standard 1 mg/day regimen in patients with endometriosis. In particular, we focus on the incidence of adverse effects related to estrogen suppression and the potential for improved treatment adherence with lower doses. Clarifying the dose-dependent relationship between pain relief and adverse effects may help to individualize hormonal therapy strategies for endometriosis.

### Study Objectives

The objective of this study is to compare the efficacy and safety profiles of dienogest 1 mg/day and 2 mg/day in patients with endometriosis-associated dysmenorrhea over a 48-week treatment period. We hypothesize that dienogest can effectively alleviate menstrual pain in these patients without significant adverse effects. Furthermore, by comparing pain improvement, the incidence of adverse effects over the 48-week period, and the occurrence of menopausal symptoms, this study aims to provide insights into optimal treatment selection for individual patients.

## Methods

### Study Design and Participants

This study is a randomized, open-label, parallel-group, dose-comparison, multicenter trial conducted at 6 centers in Japan, in accordance with the SPIRIT (Standard Protocol Items: Recommendations for Interventional Trials) 2013 guidelines (Multimedia Appendix 1). To ensure that participants have endometriosis, only patients diagnosed with ovarian endometriosis were enrolled in the study.

Eligible patients who meet the inclusion and exclusion criteria will be invited to participate in the study by the site investigators (Textbox 1).

**Eligibility criteria.**

**Inclusion criteria**

- Patients who have been diagnosed as having endometriosis (ovarian endometriotic cyst) and have not planned surgery.
- Patients with dysmenorrhea.
- Patients who plan to use dienogest for 48 weeks.
- Patients with a regular menstrual cycle and aged 20 years or older at the time of obtaining consent.
- Patients who have provided written consent to participate in this study.

**Exclusion criteria**

- Patients who have difficulty verbalizing subjective symptoms related to menstruation, etc.
- Patients currently under treatment for malignant diseases.
- Patients who have taken dienogest, low-dose estrogen progestin preparations, or other hormonal drugs within 4 weeks of obtaining consent.
- Patients in whom it has been difficult to diagnose endometriosis.
- Patients were deemed inappropriate for the study by the study investigator.

### Data Collection and Study Procedures

The study data will be collected through a combination of patient questionnaires (Multimedia Appendices 2 and 3), laboratory tests, bone density measurements, and imaging studies, including preregistration magnetic resonance imaging scans and ultrasound examinations.

Once eligibility is confirmed, study personnel will input patient information into the electronic data capture system to facilitate enrollment and randomization. Randomization using the permuted block method with a block size of 2 or 4 will be stratified based on allocation factors, such as patient age and the presence or absence of uterine adenomyosis.

Treatment will commence on the second to fifth day of the first menstrual cycle following enrollment. Patients will be randomly assigned to receive either dienogest 0.5 mg (1 mg/day group) or dienogest 1 mg (2 mg/day group), administered orally twice daily for up to 48 weeks. Follow-up visits will be scheduled at 4 weeks post enrollment and every 12 weeks thereafter.

At each follow-up visit, comprehensive assessments will be conducted, including patient questionnaires, vital sign measurements, blood tests, and ultrasound examinations to evaluate the size of ovarian endometriotic cysts. A magnetic resonance imaging scan will be performed before study enrollment to establish baseline imaging data. Pain symptoms and estrogen-deficiency symptoms will be assessed through standardized questionnaires. Dysmenorrhea-related pain will be evaluated using the visual analog scale (VAS) and a modified dysmenorrhea score based on Harada et al [20] (Table 1).

The dysmenorrhea score (total score: 0 to 6) is the sum of the subscores on the severity of pain and the use of analgesics.

Estrogen-deficiency symptoms will be assessed using the Kupperman score (Table 2).

**Table 1:** Dysmenorrhea score.

Degree		Definition	Score	
**Pain**				
	None	No pain.	0	
	Slight	Some difficulty with work, household chores, and schoolwork.	1	
	Moderate	Interference with work, housework, or schoolwork to the point of needing to lie down and take a break.	2	
	Severe	Sleeping for more than 1 day and unable to work, do housework, or study.	3	
**Painkiller usage**				
	None	None.	0	
	Slight	Used painkillers for 1 day during menstrual period.	1	
	Moderate	Used painkillers for 2 days during menstrual period.	2	
	Severe	Used painkillers for 3 days during menstrual period.	3	

**Table 2:** Kupperman score.

Symptom	Degree	Evaluation
Hot and sweaty face	3,2,1,0	4
Numbness in the limbs and loss of sensation	3,2,1,0	2
Difficulty falling asleep and waking easily	3,2,1,0	2
Easily agitated and nervous	3,2,1,0	2
Worried and depressed	3,2,1,0	2
Feeling dizzy or nauseous	3,2,1,0	1
Tire easily	3,2,1,0	1
Pain in the shoulders, knees, and knots in the hands and feet	3,2,1,0	1
Headache	3,2,1,0	1
Palpitations	3,2,1,0	1
Feels like ants crawling on the skin	3,2,1,0	1

To determine the score, multiply the “Degree of Symptomatology” (Degree) number by the “Rating Scale” number (Evaluation).

Scoring is classified as follows: mild disease: 16-20, moderate: 21-34, and severe: 35 and above. Degree is defined as strong: 3, moderate: 2, weak: 1, and none: 0.

Patients will be allowed to use analgesics for the management of dysmenorrhea-related pain as needed. Bone density assessments will be conducted twice, at the beginning and end of the study period, using dual-energy x-ray absorptiometry.

### Study Endpoints

The primary efficacy endpoint is the change in VAS from baseline to 48 weeks after administration. The secondary endpoint of effectiveness evaluation is the amount of change in the dysmenorrhea score from baseline to 48 weeks after administration. Another secondary endpoint is the rate of reduction in the size of ovarian endometriotic cysts from the baseline to 48 weeks after administration.

For safety evaluation, an important safety endpoint is the incidence of symptoms related to low estrogen (symptoms other than those listed in the Kupperman score) during the study treatment phase. Symptoms are listed in question number 5 of the questionnaire (Multimedia Appendix 3).

Other safety endpoints include the amount of change in the Kupperman score, the rate of change in bone density, and the amount of change in blood estradiol concentration from baseline to 48 weeks after administration.

### Statistical Analysis

The primary analysis involves comparing the change in VAS from baseline to 48 weeks between the dienogest 1 mg/day group and 2 mg/day group using the mean difference and its 95% CI. When the lower limit of confidence exceeds –15 (noninferiority margin), the noninferiority of the effect of the 1-mg group to the 2-mg group will be recognized.

For the secondary efficacy endpoint, each of the dienogest 1 mg/day group and the 2 mg/day group will be compared to estimate the difference and its 95% CI for the mean value of dysmenorrhea score and change in endometriotic cyst size from baseline to 48 weeks.

In the safety evaluation, the frequency of low estrogen symptoms during the study treatment period is aggregated, and a 95% CI is calculated that is based on a normal approximation of the binomial distribution. In addition, a chi-square test will be used to compare groups. The 2-sided significance level is 5%.

The frequencies of the occurrence of adverse events and side effects are calculated for the safety analysis target population. As a secondary safety analysis, each of the dienogest 1 mg/day group and 2 mg/day group will be compared to estimate the difference and its 95% CI for the mean value of bone density, Kupperman score, and estrogen concentration. Statistical analyses are performed by R (version 4.1.0; R Foundation for Statistical Computing).

### Sample Size

Based on a Japanese study [21], the mean change in VAS after 52 weeks with 1 mg dienogest was –52.1 (SD 24.13) for secondary dysmenorrhea. In the Japanese phase II dose-finding study, [19] the average change in VAS was –35.74 at 0.25 mg×2, –44.31 at 0.5 mg×2, and –50.78 at 1 mg×2 at 12 weeks. Therefore, it is assumed that at least an effect of 0.25 mg×2 or more can be expected by this test administration, and the noninferiority margin is set to 15. It is assumed that the effects of the 1 mg group and the 2 mg group are similar, and SD is assumed to be 24.13 with reference to the Japanese phase III study. [22] In the above setting, 41 cases in each group (82 cases in total) are required to achieve a power of 80% at a one-sided significance level of 2.5%. When 5% of dropout cases are added to each group, the required sample size becomes 44 cases in each group (88 cases in total).

We evaluated the incidence of low estrogen symptoms during the study treatment period as an important secondary endpoint. On the basis of the long-term use of dienogest [23], we assumed the incidence proportion for dienogest 1 mg/day and 2 mg/day to be 20% and 50%, respectively, with the chi-squared test providing more than 80% power at a 2-sided significance level of 5%. Therefore, the sample size in this study (88 cases in total) is also sufficient to assess the incidence of low estrogen symptoms.

### Ethical Considerations

The study protocol was approved by the accredited clinical research review committee of the Naoya City University Hospital Clinical Research Review Board (approval number 2021A001-23b001). Informed consent was obtained from all participants before their enrollment. Researchers must ensure that all patients who have provided written informed consent meet the inclusion criteria and do not meet any of the exclusion criteria. The study adheres to the ethical principles of the Declaration of Helsinki. All data are anonymized to protect participant privacy. No personally identifiable information is included in the dataset. In addition, stringent protective measures such as encrypted data storage and restricted access protocols have been implemented to ensure data confidentiality.

## Results

**Figure 1:**
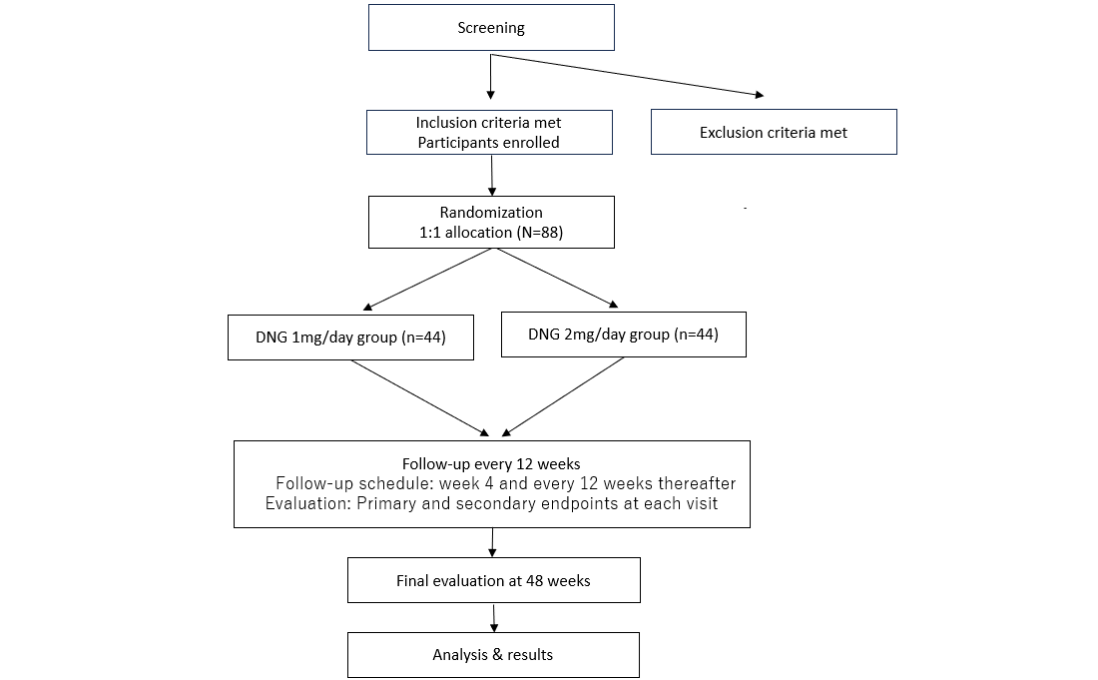
CONSORT (Consolidated Standards of Reporting Trials) flowchart. DNG: dienogest.

Participant enrollment for the trial has been completed. The first participant was enrolled in August 2021, and a total of 88 participants have been enrolled, with 44 participants allocated to each group (Figure 1). As of March 22, 2025, data collection has been finalized and is currently being fixed for analysis. Study flowchart showing the allocation of participants, in accordance with the CONSORT (Consolidated Standards of Reporting Trials) checklist (Multimedia Appendix 4).

## Discussion

### Principal Findings

This study aims to demonstrate that dienogest 0.5 mg tablets are as effective and safe as dienogest 1-mg tablets in the treatment of endometriosis. Furthermore, based on the evaluation results from this study, recommendations for the optimal use of dienogest 0.5-mg and 1-mg tablets could be proposed.

Currently, 0.5 mg dienogest tablets are approved for dysmenorrhea, but their effectiveness in improving endometriosis symptoms and lesions remains unclear.

Osuga et al [19] conducted a study comparing dienogest 1 mg/day and 2 mg/day in patients with primary dysmenorrhea and reported equivalent improvements in VAS scores between the 2 groups. However, this study did not include patients with endometriosis-related dysmenorrhea, making it insufficient to evaluate the efficacy of dienogest for endometriosis.

Another study by Osuga et al [22] compared dienogest 1 mg/day with placebo and demonstrated its efficacy in treating dysmenorrhea. However, only approximately 23% of the study population had endometriosis, which is insufficient to fully assess its effectiveness for this condition. Currently, there are no other studies comparing dienogest 0.5-mg tablets with 1-mg tablets for endometriosis, and thus, the findings of this study are expected to provide novel insights into the efficacy and safety of dienogest 0.5-mg tablets for endometriosis.

With respect to serum estrogen levels during dienogest administration, it was observed that estrogen levels did not decrease significantly with a 1 mg/day dosage compared with the placebo group. However, a dose-dependent reduction in estrogen levels was observed [19,24].

Prolonged hypoestrogenic states induced by dienogest therapy may increase the risk of adverse effects such as bone mineral density loss. Estrogen plays a crucial role in maintaining bone homeostasis by promoting osteoblast activity and inhibiting bone resorption by osteoclasts [25].

In a study by Momoeda et al [12], bone density changes were measured at 24 and 52 weeks following the administration of 2 mg/day of dienogest. There was a significant reduction in bone mass at both time points (mean –1.6%, SD 2.4%; *P*<.001; mean –1.7%, SD 2.2%; *P*<.001), though the decrease was concluded to be mild. According to Seo et al [13], after one year of administering dienogest 2 mg/day, lumbar spine bone mineral density decreased by 2.2% at 6 months and by 2.7% after 1 year compared to baseline. Furthermore, 75% (45/60) of women experienced significant bone mineral density reduction after one year. Given the high likelihood that dienogest-induced bone density reduction is correlated with estrogen levels [15], it is anticipated that the 1 mg/day group in this study will exhibit less bone loss.

While bone density reduction has been the primary focus of existing studies, other low estrogen symptoms, such as vasomotor symptoms, mood changes, and cardiovascular risks, also warrant evaluation in the context of long-term dienogest administration. Although the frequency of these symptoms is expected to be low with dienogest therapy, this study will assess the incidence and severity between the 1 mg/day and 2 mg/day groups. By analyzing these differences, this study aims to clarify whether dosage variations influence adverse effects beyond bone density reduction and provide insights into dose selection for the treatment of endometriosis-associated dysmenorrhea.

### Limitations

This study has several limitations. First, only patients with ovarian endometriotic cysts were included, excluding cases of endometriosis without ovarian involvement. This selection criterion was implemented to ensure diagnostic accuracy and maintain a homogeneous study population. However, future studies may be needed to evaluate the effects of long-term dienogest therapy in patients with other forms of endometriosis.

Second, as it was not conducted as a double-blind trial, there is a potential risk of bias in VAS and dysmenorrhea score evaluations.

This study was conducted within the framework of Japan’s health insurance system, which required specifying the prescribed drug formulation (0.5-mg or 1-mg tablet). Consequently, blinding was not feasible, and an open-label design was adopted. To minimize bias, we implemented 3 key measures. First, objective assessments, including ultrasound imaging, hormone levels, and bone density, were incorporated as primary endpoints to reduce reliance on subjective pain evaluations. Second, centralized randomization via an electronic data capture system was used to eliminate selection bias. Third, covariate adjustment for age and adenomyosis presence ensured balanced group allocation. These strategies enhance the study’s validity and reliability.

Given that endometriosis often requires long-term treatment until menopause, evaluating the efficacy and safety of extended dienogest administration is crucial. Although the observation period of this study is limited to 48 weeks, it provides valuable data, as no previous study has examined the effects of 1 mg/day beyond 12 weeks. Further research with extended observation periods is necessary to assess long-term efficacy and safety.

### Conclusions

This study will enable us to evaluate the efficacy and safety of the dienogest dosage over 48 weeks and provide a more precise and safer treatment for patients with endometriosis.

After the completion of the study, the treatment of endometriosis and assessment of side effects in enrolled patients will continue. The findings from this study will be disseminated through conference presentations and academic publications to contribute to the ongoing refinement of treatment strategies for endometriosis.

## Appendix

Multimedia Appendix 1 SPIRIT (Standard Protocol Items: Recommendations for Interventional Trials) checklist.

Multimedia Appendix 2 This figure shows the medical questionnaire for the first visit.

Multimedia Appendix 3 This figure shows the medical questionnaire for the 2nd and subsequent visits.

Multimedia Appendix 4 CONSORT (Consolidated Standards of Reporting Trials) checklist.

## Abbreviations

CONSORT: Consolidated Standards of Reporting Trials
SPIRIT: Standard Protocol Items: Recommendations for Interventional Trials
VAS: visual analog scale

## Data Availability

As this study is still ongoing, the data are not currently available for public use. However, the datasets generated or analyzed during this study are available from the corresponding author on reasonable request.
