# Supplementary figures and images for: Evaluating the Efficacy and Safety of 48-Week Low-Dose Dienogest Administration in Patients With Dysmenorrhea Caused by Endometriosis: Protocol for a Randomized, Open-Label, Parallel-Group Trial
Source: JMIR Res Protoc. 2025 May 13;14:e66246. doi: 10.2196/66246 (PMC12117269; doi:10.2196/66246)

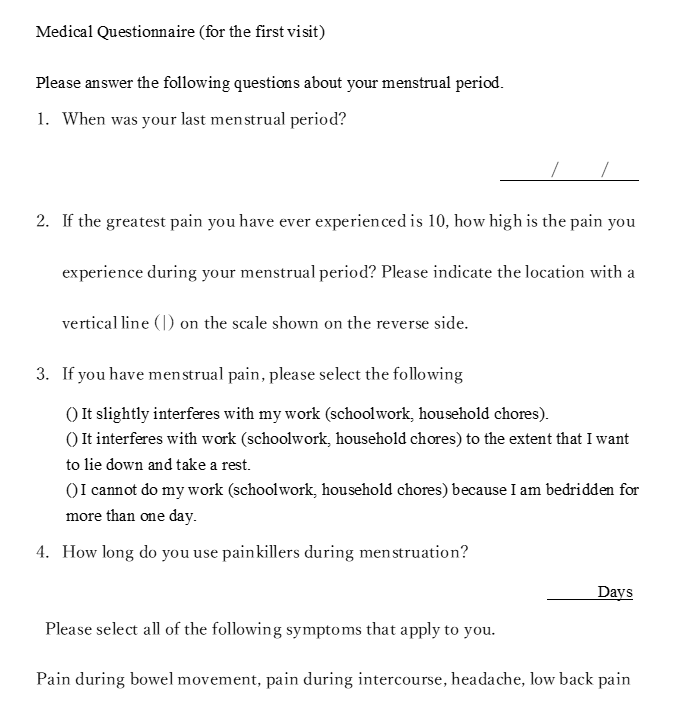

Supplement: Multimedia Appendix 2 [file resprot_v14i1e66246_app2.png]

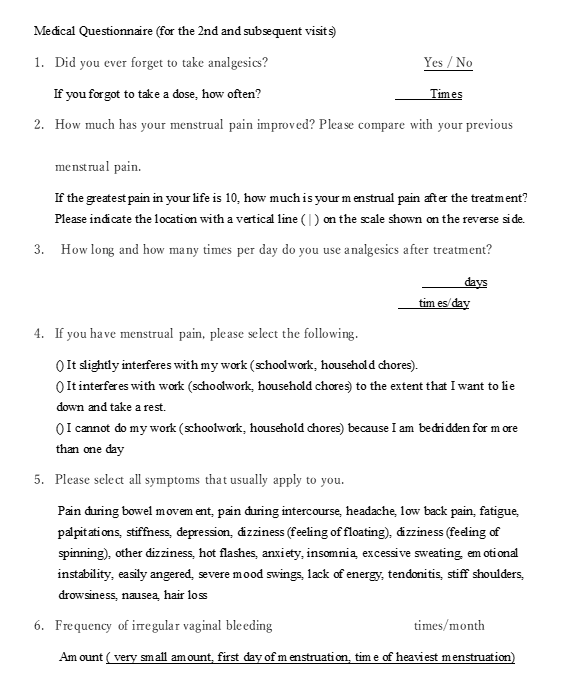

Supplement: Multimedia Appendix 3 [file resprot_v14i1e66246_app3.png]
